# Slipped capital femoral epiphysis in a 5-year-old boy with cerebral palsy on valproic acid and levetiracetam for epilepsy: a case report

**DOI:** 10.1093/jscr/rjae058

**Published:** 2024-02-13

**Authors:** Osama R Aldhafian

**Affiliations:** Department of Surgery, College of Medicine, Prince Sattam Bin Abdulaziz University, Al-Kharj, Riyadh 11942, Saudi Arabia

**Keywords:** hip pain, slipped capital femoral epiphysis, cerebral palsy, valproic acid, levetiracetam

## Abstract

This study presents a rare case of unilateral slipped capital femoral epiphysis treated surgically in a 5-year-old boy with cerebral palsy who was born at 27 weeks’ gestation and developed grade III intraventricular haemorrhage and periventricular leucomalacia and was on antiepileptic drugs, including valproic acid and levetiracetam for >3 years. The patient had no history of endocrine, renal, and significant familial diseases.

## Introduction

Slipped capital femoral epiphysis (SCFE) is the most common hip disorder among adolescents. Early and appropriate management can reduce morbidity and complications [1]. The average age of onset of SCFE is 12.7 years for boys and 11.2 years for girls; however, it is rare in children aged <10 years. SCFE has multifactorial risk factors including age, sex, duration of symptoms, race, geographic variation, and seasonal variation, indicating that genetic and environmental factors may play a role in the disease [2]. In addition, the endocrine risk factors, including hypothyroidism, growth hormone administration, renal osteodystrophy, and endocrinopathies [3–8].

Contributory biomechanical factors which may play a role in the disease, such as femoral retroversion, physeal obliquity, and obesity, lead to abnormal stress on the physis and anterolateral displacement of the femoral metaphysis away from the epiphysis [9–12].

This study aimed to describe the case of a 5-year-old independent ambulatory boy with cerebral palsy (CP) treated with antiepileptic drugs (AEDs) who was diagnosed with unilateral unstable SCFE with no endocrinopathies and was treated surgically with percutaneous *in situ* fixation.

To the best of our knowledge, no similar cases have been reported previously.

## Case report

**Figure 1:**
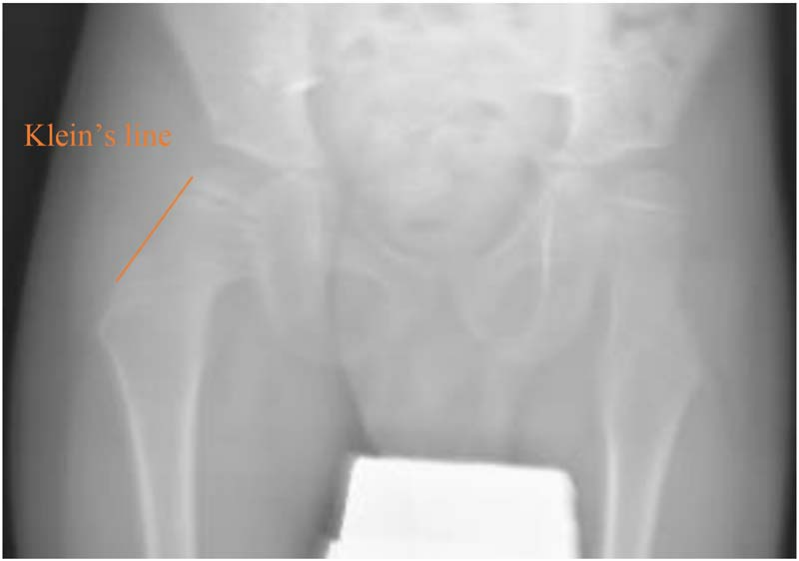
Pelvic anterior–posterior radiograph showing coxa valga deformity in the left hip.

**Figure 2:**
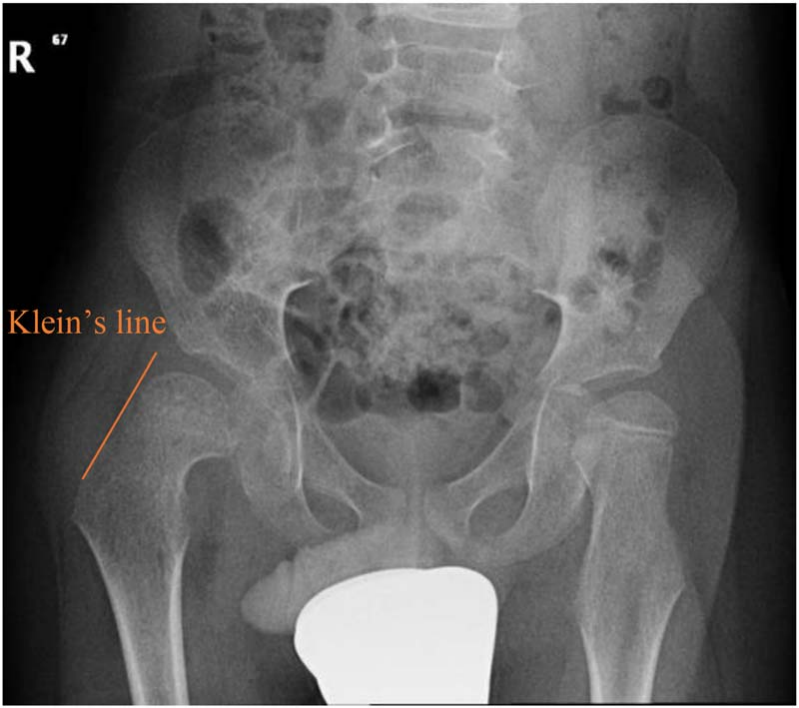
Pelvic anterior–posterior radiograph showing SCFE in the right hip, with Klein’s line not intersecting the capital femoral epiphysis

**Figure 3:**
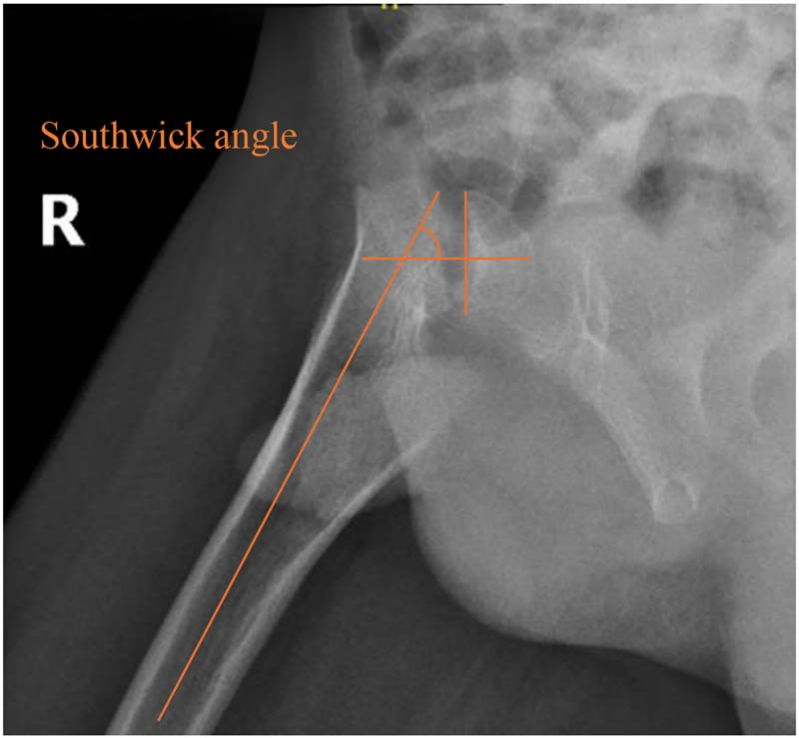
Pelvic frog-leg lateral view radiograph showing Southwick’s slip angle 50^°^ in the right hip.

**Figure 4:**
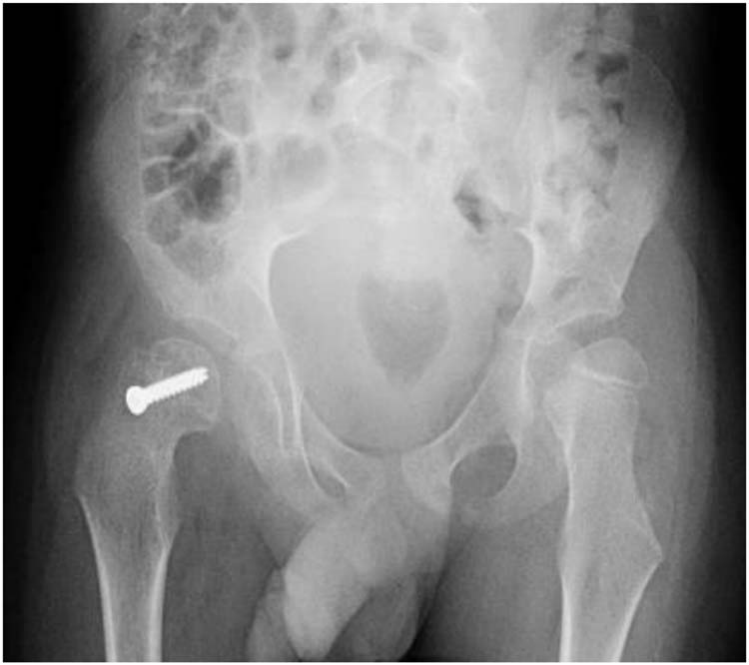
Pelvic anterior–posterior radiograph immediately after *in situ* fixation with single cannulate screw.

**Figure 5:**
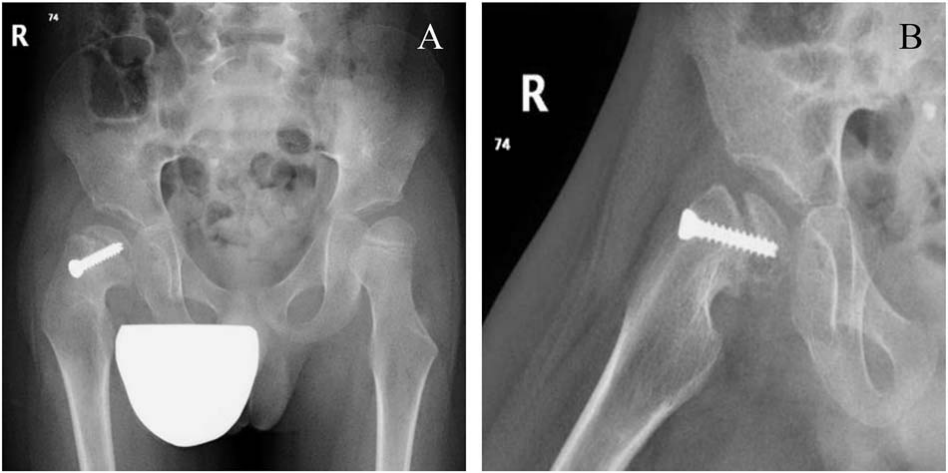
Six weeks following post-operative fixation: (A) pelvic anterior–posterior radiograph and (B) pelvic frog-leg lateral view radiograph.

**Figure 6:**
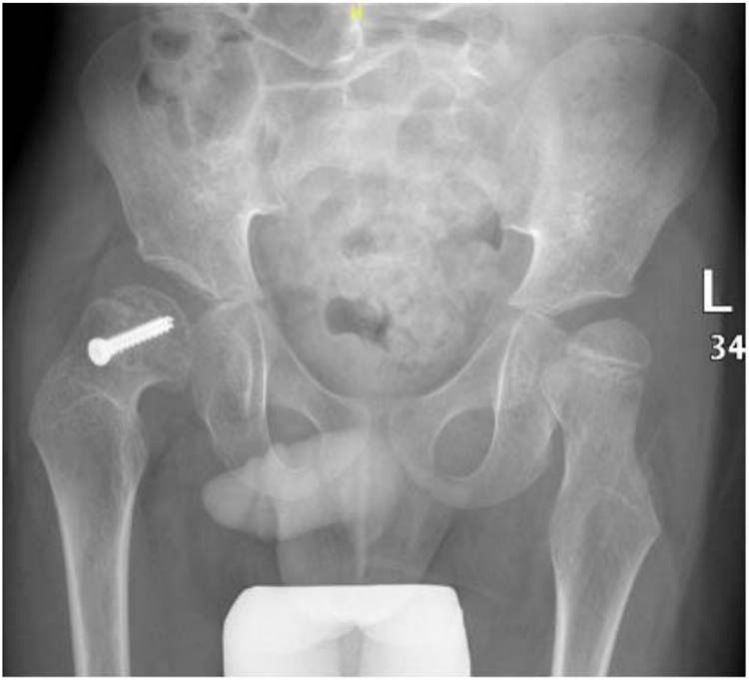
Pelvic anterior–posterior radiograph, 3 months following post-operative fixation.

**Figure 7:**
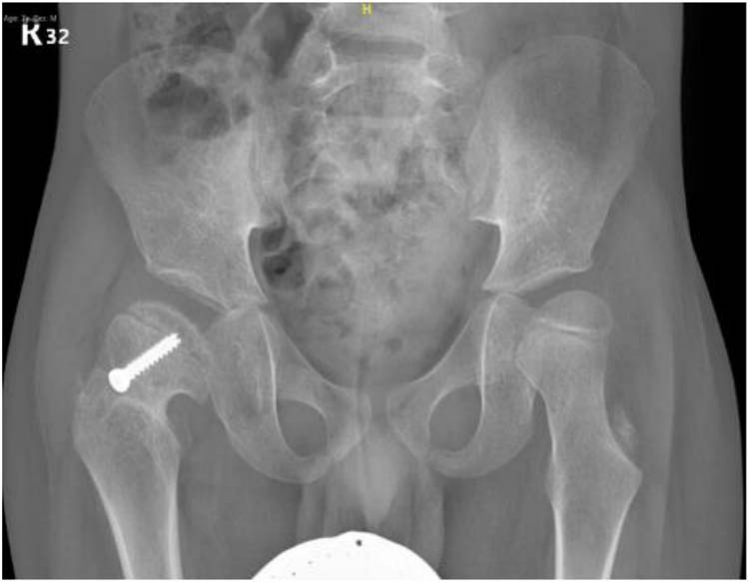
Pelvic anterior–posterior radiograph, 15 months following post-operative fixation.

**Figure 8:**
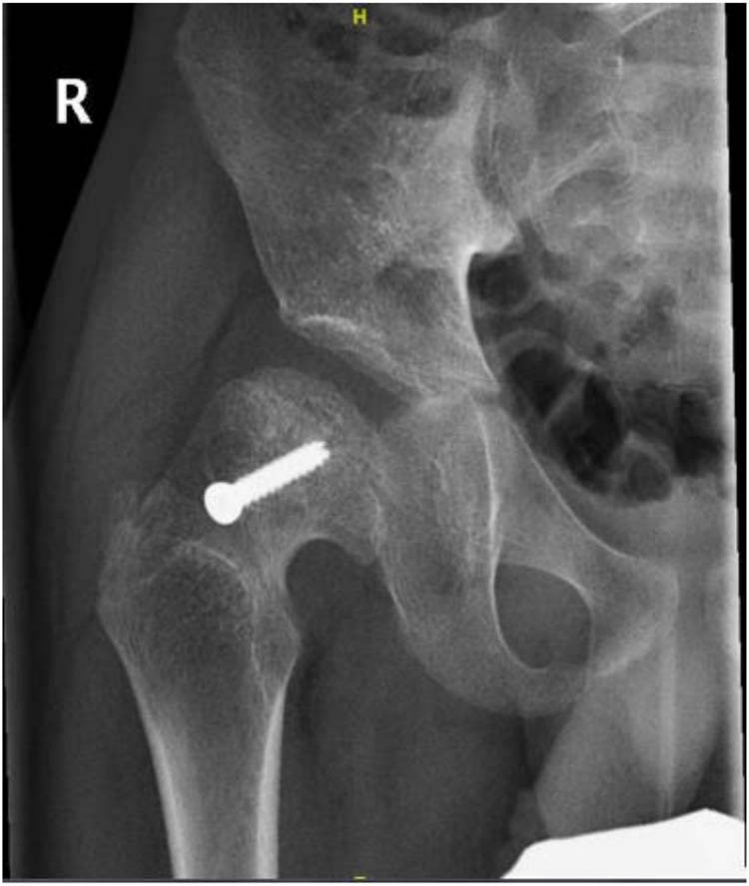
Right hip anterior–posterior radiograph, 36 months following post-operative fixation.

A 5-year-old independent ambulatory Middle Eastern boy with CP who was born preterm and developed grade III intraventricular haemorrhage and periventricular leucomalacia and was on AEDs, including valproic acid (VPA) and levetiracetam (LEV), for >3 years and was controlled over the last year (no history of seizure attack) presented to the emergency room (ER) with right hip pain and inability to bear weight for 4 weeks; the patient had no history of fever or trauma. Physical examination shows a thin, the weight is 12 kg, the height is 101 cm, vital signs within the normal range, tenderness over the right hip, and external rotation of the right hip, with restricted hip mobility. A radiological study was performed ~3 months before the patient presented to the ER for follow-up examination of a left hip coxa valgus deformity with no apparent abnormalities in the right hip (Fig. 1). Initial imaging studies conducted in the ER showed an anterior–posterior view of the pelvic radiograph, revealing Klein’s line [13] not intersecting the capital femoral epiphysis (Fig. 2), and frog-leg lateral view radiograph of the right hip (Fig. 3) confirmed SCFE and Southwick’s slip angle [13] of ~50° (moderate). Laboratory findings were clear for endocrine and renal diseases or infection, except for low vitamin D (total 25-OH Vitamin D: 43.4 nmol/L), suggesting vitamin D insufficiency. The diagnosis was confirmed with clinical and radiological studies as right-sided unstable SCFE requiring surgery. Surgical intervention was performed with percutaneous *in situ* fixation using a single fully threaded 4.5-mm cannulated screw (Fig. 4). Postsurgical rehabilitation included non-weight-bearing right lower extremities for 6 weeks. Regular follow-up with serial radiology studies showed stable fixation with no migration of screw or further slippage at 6 weeks (Fig. 5) and 3 (Fig. 6), 15 (Fig. 7), and 36 months (Fig. 8). During follow-up, a painless range of motion in the right hip was observed, with full weight-bearing and resumption of his usual activities with no complaints.

## Discussion

SCFE is the most common hip disorder among adolescents in a rapid growth phase [1]. It is rarely affect children aged <10 years. SCFE in this age group is usually due to hypothyroidism, growth hormone administration, renal osteodystrophy, and endocrinopathies [3–8]. There is no known established relationship between incidence of SCFE, epilepsy, and AEDs in the literature. The aim of this study to describe the case of a 5-year-old independent ambulatory boy with CP treated with AEDs who was diagnosed with unilateral unstable SCFE with no endocrinopathies and was treated surgically with percutaneous *in situ* fixation.

VPA is a broad-spectrum AED used for all types of seizures and syndromes. Its excellent efficacy has been demonstrated over almost 40 years of clinical experience [14, 15]. The mechanism of action of VPA remains unknown, although it has been linked to the blockade of voltage-dependent sodium channels and potentiation of GABAergic transmission [16]. Long-term use of VPA to treat epilepsy in children has been associated with bone weakness and decreased bone mineral density. Although the mechanism underlying this effect is unknown [17–19], studies have shown that paediatric patients who use VPA as a monotherapy for epilepsy have an increased risk of fractures. However, the discontinuation of VPA treatment can reduce the risk of fractures in these patients [20].

Verrotti *et al.* [21] have revealed that epilepsy and its treatment can affect bone mineralization and calcium metabolism. Various studies have shown a significant reduction in bone mineral density in patients treated with conventional (phenobarbital, carbamazepine, valproate, etc.) and novel (oxcarbazepine, gabapentin) AEDs. Despite data on the possible effects of AEDs on calcium metabolism, the mechanisms underlying these adverse effects remain unknown. Abnormalities in calcium metabolism are believed to result from the cytochrome P450 enzyme-inducing properties of some AEDs and the resultant reduction in vitamin D levels; however, the effect of several medications (e.g. VPA) cannot be readily explained by vitamin D metabolism.

LEV is a second-generation AED that has been on the market since 2000 [22]. Its mechanism of action differs structurally and functionally from other currently available AEDs, as it binds to synaptic vesicle protein 2A (SV2A) [23].

LEV is a safe and well-tolerated novel AED, and no significant drug interactions were noted between LEV and concomitant medications because of lower protein binding and no involvement of hepatic CYP isozymes [24, 25]. It has been suggested that LEV may have no harmful effects on bone strength and metabolism [26–28]. The present study shows that several predisposing factors, such as CP, epilepsy, low vitamin D, and the use of AEDs such as VPA for >3 years, may have led to SCFE. In this study, the patient was not having seizure attacks over the last year, and during the last follow up in clinic (4 weeks before present to the ER) the right hip radiology study showed no obvious hip abnormality. Low vitamin D level might be explained by cytochrome P450 enzyme-inducing properties of some AEDs and the resultant reduction in vitamin D levels as reported earlier. In conclusion, with these predisposing factors, it can’t be linked to any of CP, epilepsy, or AEDs are the cause of SCFE in this patient. Further studies are required to investigate this relationship and to study the histopathological effects of AEDs on proximal femoral bone physis.
